# Si*x*-Axis, Physiological Activity Profiles Create a More Challenging Cellular Environment in the Intervertebral Disc Compared to Single-Axis Loading

**DOI:** 10.1021/acsbiomaterials.4c01773

**Published:** 2025-04-23

**Authors:** Daniela Lazaro-Pacheco, Isabelle Ebisch, Justin Cooper-White, Timothy P. Holsgrove

**Affiliations:** 1 Department of Engineering, Faculty of Environment, Science and Economy, University of Exeter, Harrison Building, Streatham Campus, North Park Road, Exeter EX4 4QF, U.K.; 2 School of Chemical Engineering, The University of Queensland, Brisbane 4072, Australia; 3 The UQ Centre in Stem Cell Ageing and Regenerative Engineering (StemCARE), Australian Institute for Bioengineering and Nanotechnology, The University of Queensland, Brisbane 4072, Australia

**Keywords:** intervertebral disc, si*x*-axis loading, physiological loading, complex loading, biomechanics, bioreactor, cell viability

## Abstract

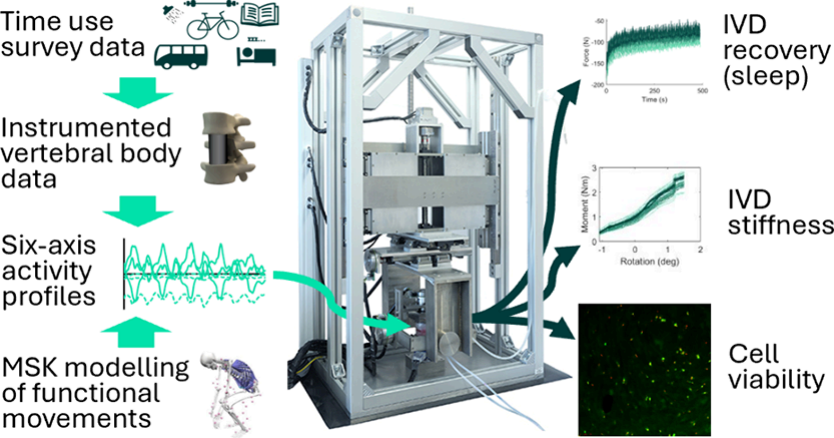

Bioreactors provide a valuable way to explore interactions between the mechanical and biological environments of the intervertebral disc (IVD), but the replication of ecologically valid loading protocols is a huge challenge. The aim of this study was to address this through the combination of time use survey data and six-axis load data from *in vivo* measurements during functional movements and activities of daily living to create population-based activity profiles, which were employed using a unique si*x*-axis bioreactor and a whole-organ bovine tail IVD model. The results of the study show that six-axis activity profiles create a more challenging environment compared to single-axis loading or unloaded controls, resulting in lower cell viability in both the nucleus pulposus and annulus fibrosus regions of the IVD. Additionally, the six-axis activity profile representing a more active lifestyle led to an even lower cell viability in the annulus fibrosus, which may be due to the increased strains in this region of the IVD during activities of daily living. These findings highlight the importance of considering a wide range of activities and lifestyles in the development and evaluation of regenerative therapies and preventative interventions for IVD, if they are to be successfully translated to the clinical setting.

## Introduction

Low back pain is the leading global cause of years lived with disability,^1^ and IVD degeneration is commonly associated with pain.^2−4^ The IVD comprises a gel-like central nucleus pulposus (NP), surrounded by multiple highly orientated collagenous layers that form the annulus fibrosus (AF), with cartilaginous end plates connecting the NP and AF to the adjacent vertebrae. This structure provides the ability to withstand high compressive loads and allows motion in all six degrees of freedom, providing the ability to bend and twist in multiple planes. The IVD is also the largest avascular structure in the human body, and the necessary nutrition and metabolite removal that dictates cell viability and IVD homeostasis occurs primarily via diffusion to and from the peripheral blood supply.^5^ However, the convective transport of nutrients and metabolites also occurs as a result of IVD loading, and this is significantly influenced by the rate of loading and level of IVD degeneration.^6^

This leads to the biomechanics, cells, and extracellular matrix (ECM) of the IVD all being linked, with disruption to any one having the potential to create a vicious cycle of degeneration.^7^ Whole-organ IVD culture studies provide a valuable way to investigate the interaction between the biomechanical and biochemical environments of the IVD, to understand more about the development and progression of IVD degeneration and to evaluate potential interventions.

Whole-organ IVD culture studies that have integrated mechanical loading have demonstrated the significant impact of different loading conditions on cell viability, gene expression, and IVD homeostasis,^8^ and these systems have also been used to investigate the effects of overloading,^9^ needle puncture and biopsy punch injuries,^10,11^ enzymatic degeneration,^12^ and cell injection therapy.^13^ However, the majority of whole-organ IVD culture studies have applied loading in axial compression only,^9,10,12−19^ or combined axial compression and axial rotation,^11,20−22^ with an extremely limited number of studies that have applied bending^23^ or multiaxis loading including bending^24^ over multiday culture periods. Additionally, many previous studies have applied loading at rates, magnitudes, and durations below physiological levels.^25^ Combined cyclic compression and torsion (CCT) has been shown to lead to a significant and substantial reduction in cell viability in the NP combined with significantly upregulated anabolic, catabolic, and proinflammatory gene expression compared to single-axis cyclic loading in either axial compression or axial torsion, or unloaded controls.^21^ This highlights the importance of considering the loading regime used in whole-organ IVD culture studies and justifies previous calls to integrate greater degrees of freedom and more physiologically relevant loading regimes to bioreactor systems.^8,26,27^ However, the use of complex si*x*-axis loading regimes to simulate activities of daily living (ADLs) in IVD cultures remains unexplored.

The expansion of bioreactor systems to include multiaxis loading would also provide the ability to complete physiologically relevant biomechanical testing of IVDs during the culture period, which is not possible in isolated cell studies, bioreactors with limited loading axes, or *in vivo* studies. Many IVD culture studies do not include biomechanical evaluation, though axial stiffness and disc height changes over the culture period have been measured.^9,18^ Integrating functional biomechanical tests in all three planes of motion at multiple time points during IVD culture studies would provide a more complete picture of the interaction between biomechanics, cells, and the ECM. This integrated approach would allow the advanced preclinical testing of treatments and therapies for pathologies such as degenerative disc disease prior to *in vivo* animal studies and first-in-human trials, thus contributing to the 3R principles of animals in research.

Therefore, the aim of this study was to exploit recent advances in spine test systems and loading protocols^28,29^ to apply population-based, six-axis activity profiles to IVD organ cultures for the first time, and compare the cell viability with an equivalent single-axis activity profile and unloaded controls. A further aim was to evaluate the biomechanical parameters throughout the test period. These combined aims have the overall objective of demonstrating the capability of a next-generation bioreactor to enhance our understanding of how the mechanical environment influences IVD health.

## Materials and Methods

### Specimen Preparation and the Biochamber Culture System

**Figure 1:**
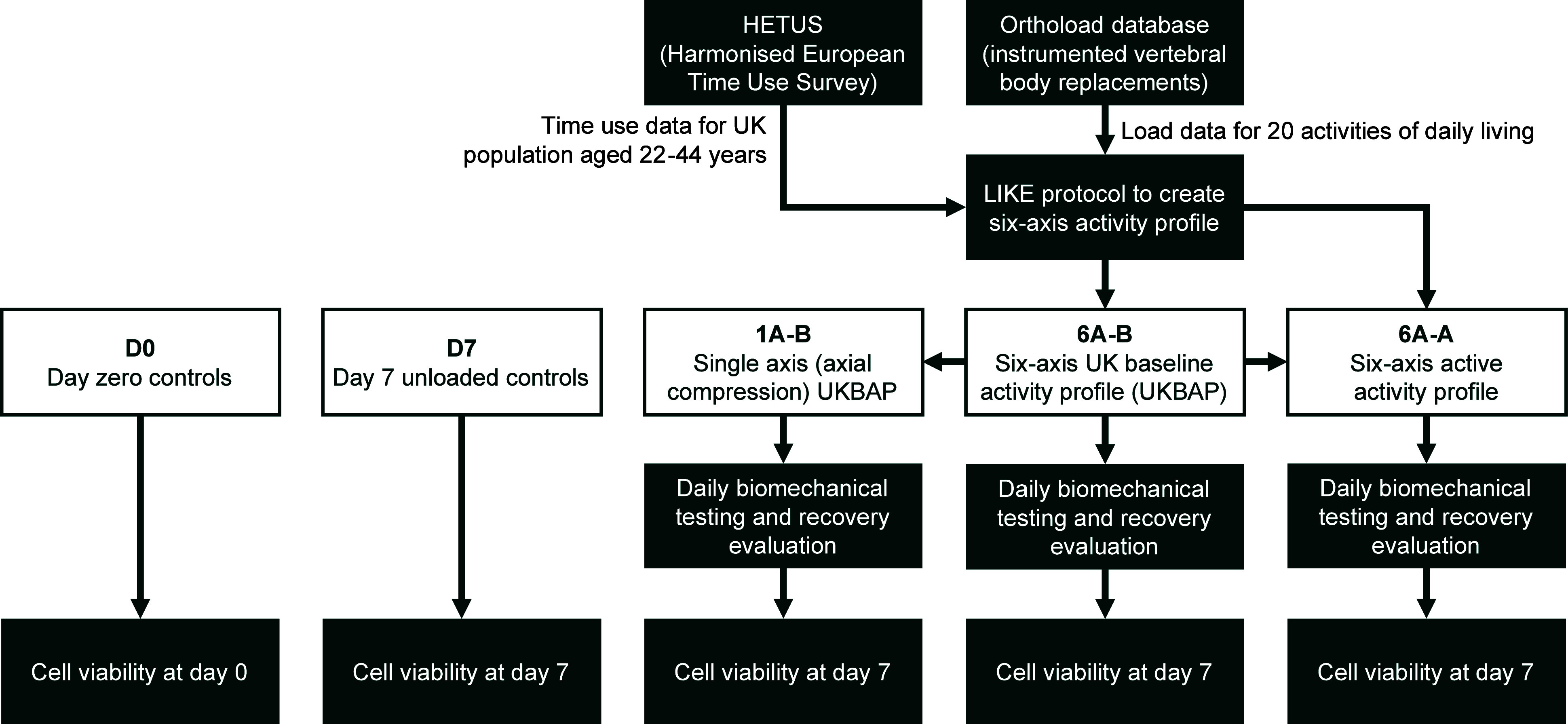
Flowchart showing the five study test groups (*n* = 4 for each group). Cell viability was measured in day zero controls on the day of acquisition (D0) and unloaded controls at day 7 (D7). HETUS and Orthoload data were used to develop a six-axis UK baseline activity profile (6A-B) using the previously developed LIKE protocol from which single-axis baseline (1A-B) and six-axis active (6A-A) activity profiles were derived. Activity profiles were applied to specimens 24 h a day for 7 days, with biomechanical tests completed three times a day, recovery during sleep evaluated daily, and cell viability measured following the end of testing on day 7.

Bovine caudal specimens were acquired from a local abattoir and processed within 3 h postmortem to maintain cellular integrity for subsequent organ culture. Specimens were assigned to one of five groups (each *n* = 4): day 0 controls (D0), day 7 unloaded controls (D7), single-axis UK baseline activity profile, six-axis UK baseline activity profile, and six-axis active activity profile (Figure 1).

For the day 0 (*n* = 4) and day 7 (*n* = 4) unloaded specimens, two tails were used for each group, with two disc levels harvested per tail (Cx1–2 and Cx2–3). For tests on the six-axis bioreactor (*n* = 12), the Cx1–2 level was harvested from 12 tails. Cattle age and sex were not recorded but would generally be of the typical UK slaughter age of 22–23 months for both male and female beef cattle. The mean ± standard deviation lateral (*D*) and anteroposterior (*d*) diameters for the three groups were as follows: 1A-B: *D* = 28.32 ± 0.56 mm, *d* = 27.72 ± 0.80 mm, 6A-B: *D* = 27.95 ± 2.18 mm, *d* = 25.30 ± 2.25 mm, 6A-A: *D* = 25.64 ± 1.89 mm, *d* = 25.58 ± 1.86 mm.

**Figure 2:**
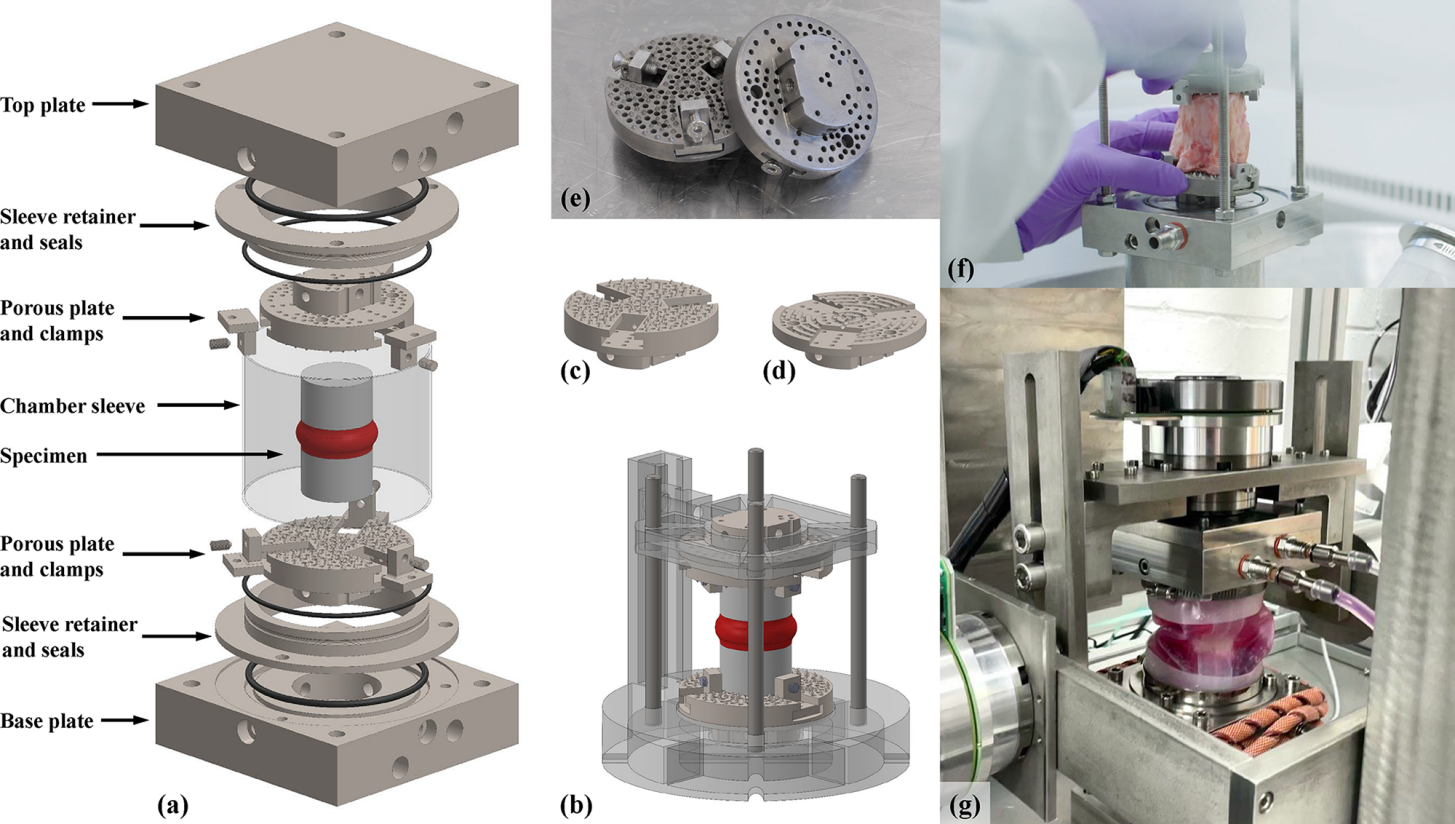
Biochamber assembly used in the six-axis bioreactor. (a) Exploded isometric view of the design showing the top and baseplates with media inlet/outlet, retainer rings and seals, the spiked porous plates and clamps to fix the specimen via the vertebral bodies, and the flexible biochamber sleeve. (b) Alignment fixture used to secure the specimen to the superior and inferior porous plates prior to mounting the plates to the top and baseplates and within the flexible biochamber sleeve. (c) Porous plate design with spikes for specimen fixation, T-slots for clamps to provide additional fixation, and holes to allow the free circulation of culture media during testing. (d) Cross-sectional view of the porous plate to show the interlinked internal channels, which ensure free flow of media to and around the specimen. (e) 3D-printed stainless steel porous plates with clamps. (f) Specimen fixed to the porous plates being positioned onto the baseplate prior to the assembly of the biochamber sleeve, and retainer rings and top plate. (g) Specimen mounted in the biochamber, which is itself mounted on the six-axis bioreactor, with media circulated via an external pump, and heating coils used to maintain media and chamber temperature at 37 °C.

Specimen processes and ligaments were removed, and 10 mm of vertebral body adjacent to the IVD was retained to secure specimens within the biochamber. D0 controls were processed for cell viability immediately after dissection. All other groups were cultured for 7 days using the Prime Growth isolation/neutralization/culture media system (Primegrowth, Wisent, Canada)^30^ to prevent both blood clot interference with fluid dynamics and to preserve specimen integrity throughout culture testing. After preparation, D7 specimens were cultured in sterile specimen containers adapted with a PVDF 0.22 μm membrane filters in an incubator at 37 °C and 5% CO_2_. Loaded specimens, once vertebral bodies were affixed in the biochamber via spiked porous plates and radial clamping, were mounted onto a custom six-axis spine simulator (Figure 2). During testing, the culture medium was circulated through the chamber using a peristaltic pump to mimic the dynamic fluid flow around the IVD *in vivo*. The biochamber was maintained at 37 °C via heating coils wrapped around the base of the chamber and around the base of the culture reservoir, and the CO_2_ level was maintained at 5%.

### IVD Loading Profiles

The United Kingdom baseline activity profile (UKBAP) was developed using the Harmonised European Time Use Survey (HETUS) for a UK cohort aged 24–44 years^31^ to reflect the increase in back pain and disc degeneration onset in this population group; a single country-level group was chosen to minimize behavioral disparities arising from cultural differences. HETUS provided a structured outline of 5 main and 40 subactivity categories. Using the *in vivo* six-axis data from instrumented vertebral body replacements available via the Orthoload database,^32^ 20 activities were selected and adjusted to match the HETUS-derived daily activity profile, producing a detailed UKBAP.^28^ This activity profile was applied to replicate spinal movements and loading of the IVD during ADLs, with adaptations to the profile made to evaluate the effect of an equivalent single-axis activity profile limited to axial compression alone, and a more active profile. All loaded IVD specimens underwent continuous loading for 7 days using these profiles with daily biomechanical testing and load recovery evaluation. Once specimens were mounted on the six-axis test system, the position in all axes was adjusted to achieve zero load, and the position then offset to zero, from which test profiles were applied.

For all tests completed on the six-axis bioreactor, the 7-day activity profile created using the LIKE protocol^28^ was converted to a drive file. The drive file was uploaded to the dSPACE controller, which provides the overall control for the six-axis bioreactor (Figure S1, suppl.). The drive file includes time and desired signals (load or position) for each axis with a sample rate of 100 Hz, which allows all axes to be controlled synchronously. The drive file also includes trigger channels that are used by the dSPACE system to trigger data acquisition simultaneously across all axes. The single-axis UKBAP (1A-B) group was completed in load control after the six-axis UKBAP testing was completed using the average axial compressive loads applied to specimens in the six-axis UKBAP group (mean ± standard deviation compressive force of 173 ± 92 N during the day and 19 ± 5 N during the recovery/sleep period). All other axes were held in a fixed position. By matching the axial loading of the si*x*-axis UKBAP group, it was possible to directly compare the cell viability resulting from the replication of the same ADLs in a simplified single-axis protocol with the full six-axis protocol.

**Figure 3:**
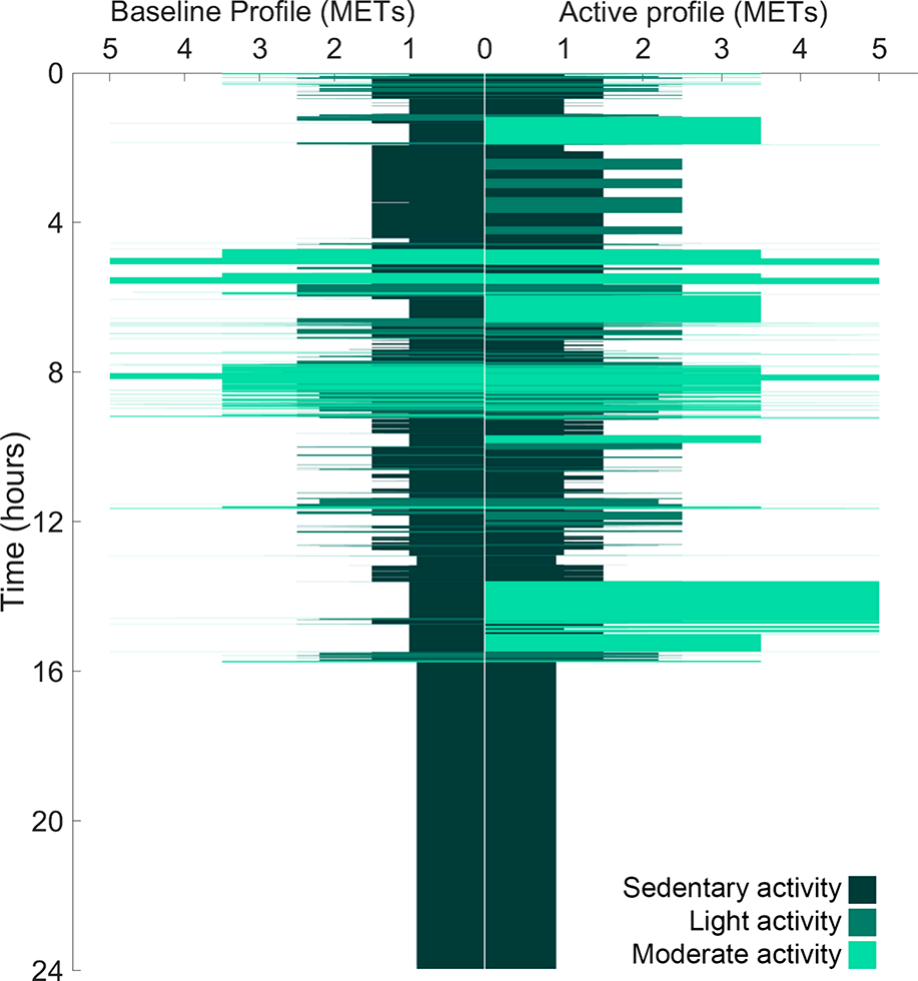
Comparison of UKBAP used for 6A-B test group (left) and active profile used for the 6A-A test group (right). Profiles were developed from HETUS-based 24 h activity profiles for a UK population aged 24–44 years, categorized via the METs^33^ as low-intensity (sedentary) activity (METs < 1.5), light-intensity activity (METs = 1.5–3), and moderate-intensity activity (METs = 3–6).

The six-axis UKBAP (6A-B) group consisted of a complex loading regime based on the LIKE protocol,^28^ which used the UKBAP to create a 24 h physiological activity profile (Figure 3), including daily disc height changes via diurnal correction curves, which was applied via six-axis kinematic control: anterior–posterior shear (Tx), lateral shear (Ty), axial compression (Tz), lateral bending (Rx), flexion-extension (Ry), and axial rotation (Rz). This complex, six-axis loading strategy aimed to closely mimic the dynamic *in vivo* mechanical environment of the IVD. This UKBAP represents a relatively sedentary lifestyle reflective of HETUS data for the UK population aged 24–44 years.

The six-axis active activity profile (6A-A) group was designed to represent an active lifestyle in six axes. To achieve this, the UKBAP was adjusted by using the metabolic equivalents of task (METs) of different ADLs^33^ and replacing relevant low-intensity (sedentary) activities (METs < 1.5) with light/moderate-intensity activities (METs = 1.5–6) (Figure 3). For example, commuting by car was replaced by cycling, or watching TV was replaced with an exercise workout. Sleep time and sleep movements (e.g., moving between sleeping positions such as supine to side lying) were unchanged between profiles. As a result, the daily sedentary time of 19.35 h in the 6A-B group was reduced to 14.78 h in the 6A-A group, and the total daily MET minutes increased from 2086 to 2788. The 6A-A profile was applied to specimens using the LIKE protocol strategy in the same way as the 6A-B profile, including 6A-A specific diurnal correction curves to account for increased fluctuation in disc height due to the increased intensity of activities in the 6A-A profile.

### Cell Viability

Cell viability in all groups was determined using the same protocol, which was completed in D0 specimens immediately after dissection on the day of acquisition and after 7 days of culture in D7, 1A-B, 6A-B, and 6A-A groups. Each disc was dissected from the vertebral bodies, halved, and NP and AF segments (3 mm) were excised (Figure S2, suppl.). The tissue was incubated at room temperature for 2 h with a LIVE/DEAD Viability/Cytotoxicity Assay Kit (Molecular Probes, USA), which was prepared with the Primegrowth culture media. Postincubation, tissues were flash-frozen in liquid nitrogen; 30 μm sections were cut using a cryomicrotome, mounted on glass slides (VECTASHIELD, Vector Laboratories), and imaged with an inverted Leica SP5 confocal microscope using 488 and 543 nm lasers. Six images per tissue region (NP and AF) were captured from at least two nonconsecutive sections to ensure representative sampling (Figure S2, suppl.). Cell viability was defined as the percentage of live cells with respect to the total number of cells on each image, and this was averaged across the six images per tissue type to provide cell viability for each specimen in the NP and AF.

### Biomechanical Testing

Biomechanical tests were completed in loaded groups three times a day during the 7-day culture period, chosen to be equivalent to the state after waking up, at noon, and before bed. The tests used the si*x*-axis kinematics obtained using an *in vivo*, *in silico*, *in vitro* pipeline.^29^ This provided a means to complete tests during functional movements based on the *in vivo* data of healthy participants. Each biomechanical test comprised four cycles of flexion with a straight back (FSB), flexion with a bent back (FBB), lateral bending (LB), and axial rotation (AR). During testing, the load and position data was acquired at 100 Hz, and the data from the fourth cycle of each functional movement was used for the analysis. Specimen stiffness was determined by a linear regression model. The rotational stiffness was calculated for the relevant functional movements in the six-axis test groups (6A-B and 6A-A) along with the effective axial stiffness during the functional movements. It should be noted that as each test replicated the kinematics derived from the six-axis loading of functional movements, the results are not pure stiffness measures in rotational axes and axial compression but instead a more general stiffness measure in the primary axes and in compression during each movement, which was then evaluated for change over the course of the testing period. As the loading in the single-axis test group (1A-B) was limited to axial compression alone, the axial stiffness was calculated for the uniaxial equivalents of the above functional movements (uFSB, uFBB, uLB, and uAR).

The 8 h 20 min profile segment representing sleep was also recorded, with load and position data acquired at 0.017 Hz (1 measurement per minute). The consistency of the 1A-B, 6A-B, and 6A-A groups was used to evaluate IVD load and recovery and the effect that the different activity profiles had on the load and recovery over the 7-day culture period. The T*z*-axis parameters at the start (defined as 5 min after the beginning) and end (1 min before the end) of the sleep period were identified. The start parameter was used to identify consistency at the start of the sleep period each day, and the difference between the start and end parameters was used to calculate the IVD recovery parameters for each day. It is of note that as the 1A-B group tests were completed in load control, the sleep parameters were based on the position in the T*z*-axis and change in Tz position over the sleep period. Conversely, as the 6A-B and 6A-A group tests were completed in six-axis kinematic control, the sleep parameters were based on the force in the T*z*-axis and the change in Tz force over the sleep period.

### Statistical Analyses

As there would be a period of equilibration and stabilization following the specimens being placed within the biochamber and being subjected to the activity profiles after the dissection and preparation process, the biomechanical test and sleep parameters of day 1 were not included in the statistical analyses. Due to the variation between specimens and differences in control methods between single- and six-axis tests, biomechanical and sleep analyses were also limited to evaluating within-group changes in parameters over the test period rather than direct comparisons between groups. All statistical analyses were completed in the PRISM Software (Graphpad, La Jolla, USA), with a significance value of 0.05.

The cell viability in the NP and AF of all groups was compared using one-way ANOVA, and in cases of significance, Tukey's multiple-comparison post hoc analyses were completed to compare D0, D7, 1A-B, 6A-B, and 6A-A groups. Stiffness parameters were analyzed using repeated-measures two-way ANOVA with time of day and day factors to identify whether the stiffness was different at different times of day, and whether there were significant changes in stiffness parameters over the course of the culture period. In cases where time of day was a significant factor, Tukey's post hoc analyses were completed to compare individual times of day for each test day. In cases where day was a significant factor, Dunnett's post hoc analyses were completed for each time of day to compare parameters from days 3 to 7 with day 2. Sleep parameters were analyzed using the repeated-measures one-way ANOVA and in cases of significance a Dunnett multiple-comparison post hoc test used to compare parameters from days 3 to 7 with day 2.

## Results

### Cell Viability

**Figure 4:**
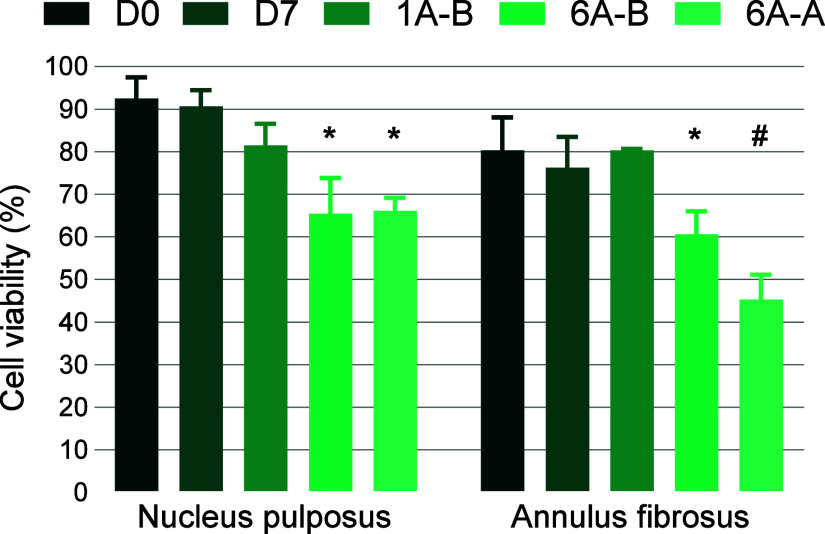
Cell viability in unloaded controls (D0 and D7), single-axis UKBAP (1A-B), six-axis UKBAP (6A-B), and six-axis active activity profile (6A-A). *Significant difference with respect to D0, D7, and 1A-B groups. #Significant difference with respect to D0, D7, 1A-B, and 6A-B groups.

There were significant differences in cell viability between groups in both the NP and AF (*p* < 0.001) (Figure 4). The cell viability in D0 controls was high in both the NP (mean ± standard deviation, 92.5 ± 5.0%) and AF (80.3 ± 7.8%), demonstrating that the specimen acquisition and preparation methods could maintain good cell viability prior to culture. The D7 controls also demonstrated that the culture protocols maintained good cell viability over the test period in both the NP (90.1 ± 3.8%) and AF (76.2 ± 7.3%), and no significant differences were observed between the D0 and D7 controls (*p* > 0.862).

The single-axis (1A-B) activity profile led to the highest cell viability of the loaded groups in both the NP (81.5 ± 5.0%) and the AF (80.3 ± 0.36%), and there were no significant differences between the 1A-B group and the D0 or D7 controls in either the NP (*p* > 0.074) or AF (*p* > 0.860). However, both six-axis (6A-B and 6A-A) activity profiles resulted in significantly lower cell viability compared to control and single-axis loading groups in both the NP (*p* < 0.008) and the AF (*p* < 0.015) (Figure 4). In the 6A-B group, the cell viability after 7 days was 65.4 ± 8.4% and 60.6 ± 5.4% in the NP and AF, respectively. In the 6A-A group, replicating a more active profile, the cell viability was 66.1 ± 3.1 and 45.3 ± 5.8% in the NP and AF respectively, and the cell viability in the AF region of this group was also significantly lower than the more sedentary 6A-B profile (*p* < 0.018).

### Biomechanics

**Figure 5:**
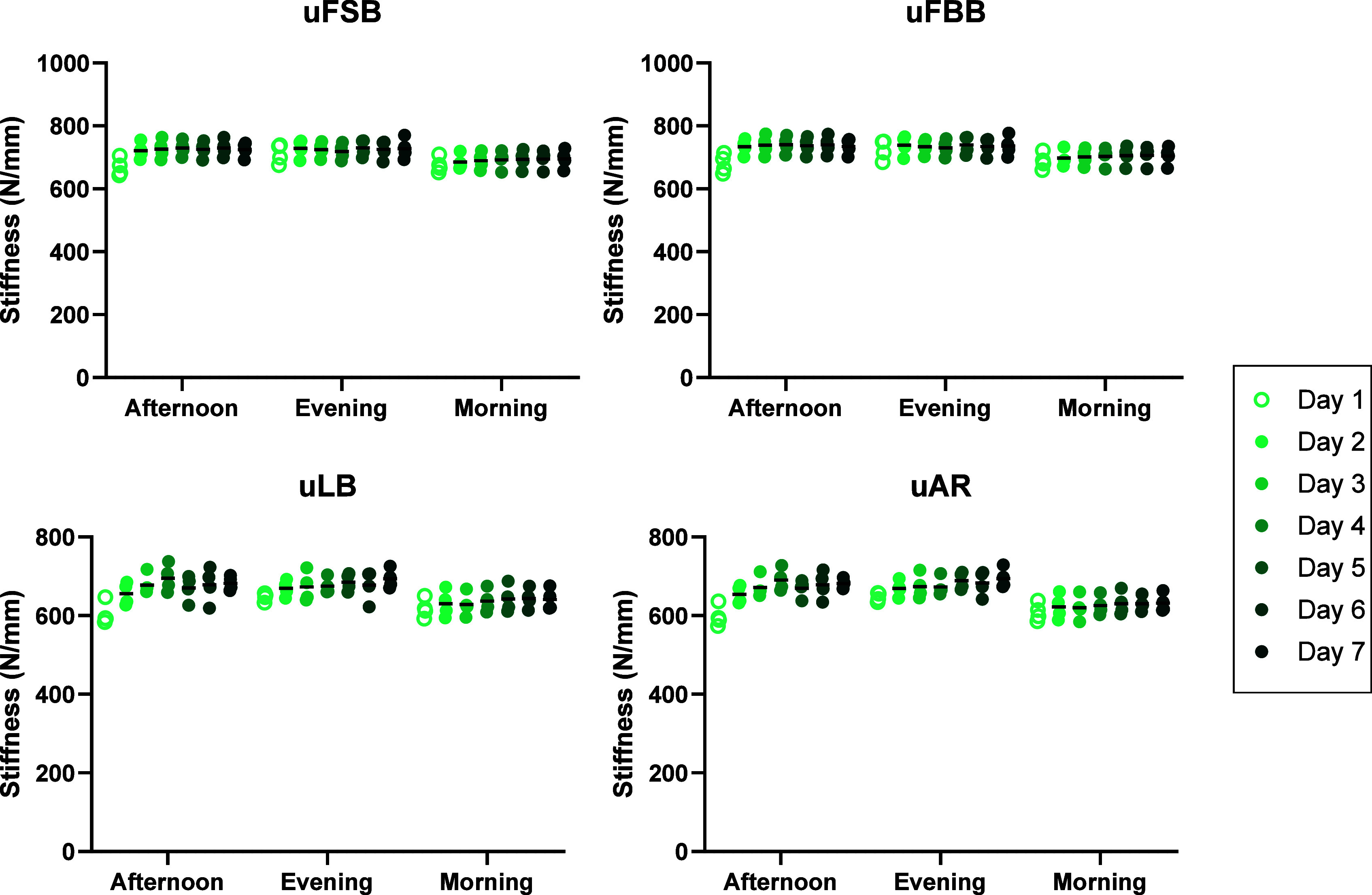
Axial stiffness in the single-axis UKBAP (1A-B) group during the four functional movement biomechanical tests across the 7 days of testing: uniaxial flexion with straight back (uFSB), uniaxial flexion with bent back (uFBB), uniaxial lateral bending (uLB), and uniaxial axial rotation (uAR). Lines denote the mean stiffness value. It should be noted that as the 1A-B tests were completed using axial compression alone, the functional movement tests only replicated the axial compression aspect of each movement, hence the uniaxial (u) prefix to differentiate it from the six-axis functional movement tests of the six-axis UKBAP (6A-B) and six-axis active activity profile (6A-A) groups.

The single-axis (1A-B) tests, which were limited to axial compression using load control, resulted in low variability across the group (Figure 5). The stiffness measured across the four functional tests was similar, as these tests were all limited to axial compression. There was no significant effect of test day on the 1A-B stiffness (*p* > 0.220) (Table 1, suppl.), but time of day was significant in all test days (*p* < 0.014), with post hoc tests revealing that the majority of these differences occurred between the morning and afternoon (17/31, 55%) and morning and evening (12/31, 39%) (Table 2, suppl.).

**Figure 6:**
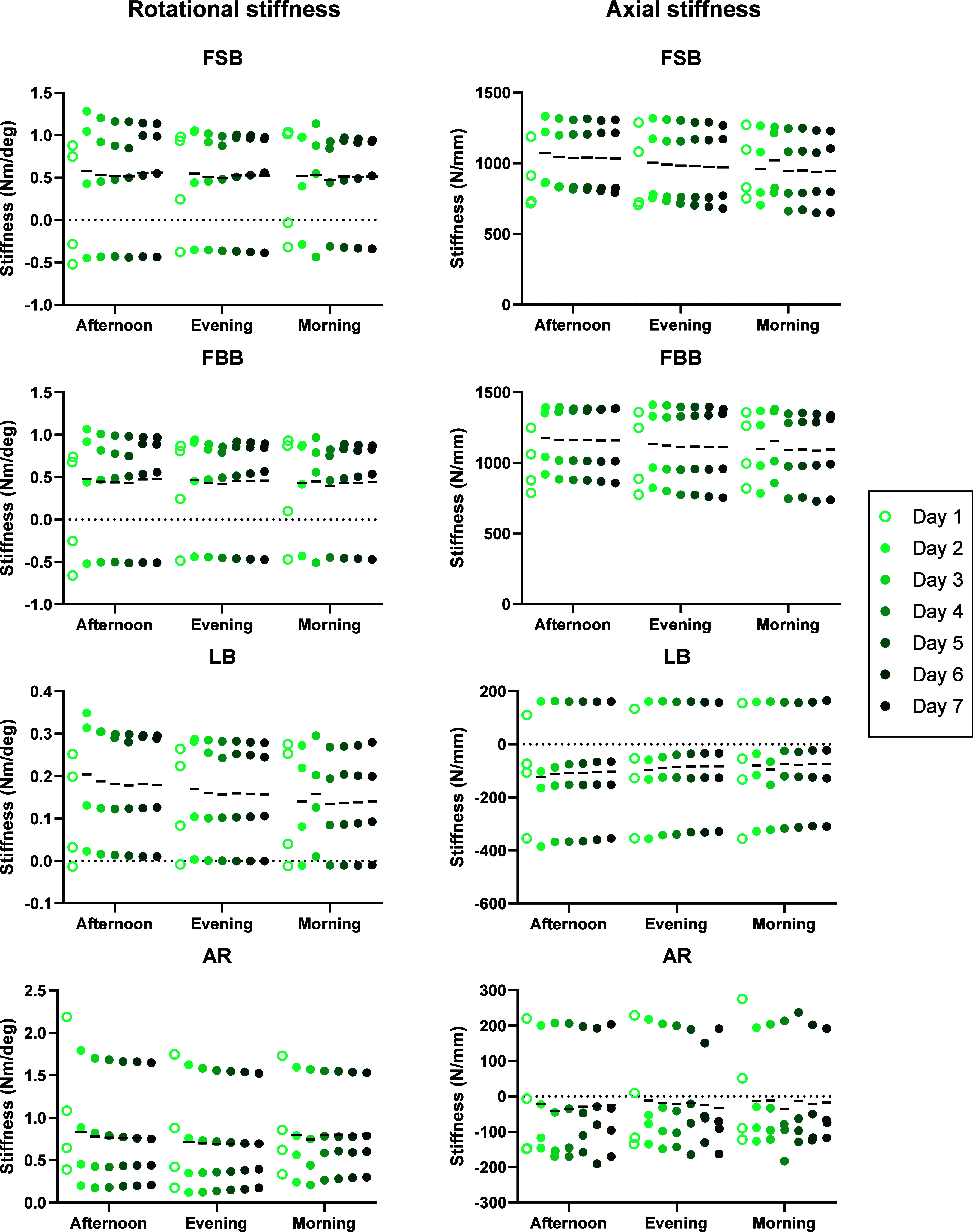
Rotational (left column) and effective axial stiffness (right column) in the six-axis UKBAP (6A-B) group during the four functional movement biomechanical tests across the 7 days of testing: flexion with straight back (FSB, row 1), flexion with a bent back (FBB, row 2), lateral bending (LB, row 3), and axial rotation (AR, row 4). Lines denote the mean stiffness value.

**Figure 7:**
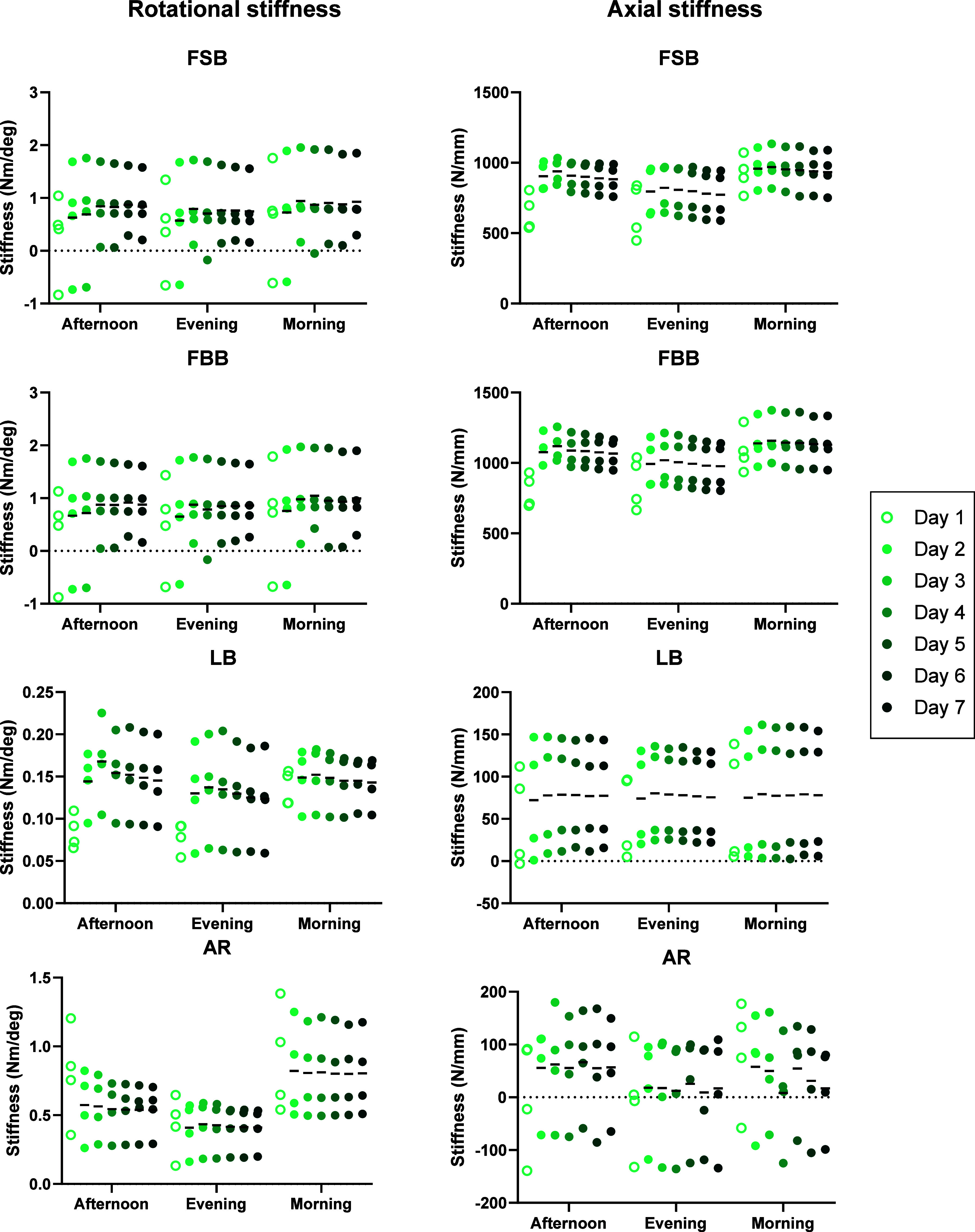
Rotational (left column) and effective axial stiffness (right column) in the six-axis active activity profile (6A-A) group during the four functional movement biomechanical tests across the 7 days of testing: FSB (row 1), FBB (row 2), LB (row 3), and AR (row 4). Lines denote the mean stiffness value.

The biomechanical tests in the six-axis (6A-B and 6A-A) groups, which simulated the more complex six-axis kinematics of functional movements, led to more varied responses across the specimens (Figure 6 and 7, respectively). There were no significant effects of test day on the rotational or axial stiffness in the 6A-B group (Table 1, suppl.), but time of day was a significant factor in the axial stiffness of the FSB test between the morning and afternoon on day 2 (*p* = 0.042) (Table 2, suppl.). Time of day was also a significant factor in the 6A-A group: rotational stiffness during AR (*p* = 0.032), axial stiffness during FSB (*p* = 0.028), and axial stiffness during FBB (*p* = 0.028). In rotational AR stiffness, post hoc analyses identified that this was due to differences between morning and evening (4/8, 50%) and between afternoon and evening (4/8, 50%) spread across the testing period (Table 2, suppl.). In the axial stiffness of FSB and FBB tests, although time of day was identified as a significant overall factor, no specific times of day were identified as significant during post hoc analyses (*p* > 0.060). The test day was a significant factor in the rotational stiffness of LB in the 6A-A group (*p* = 0.017); however, there were no significant differences between specific days identified through post hoc analyses (*p* > 0.087) (Table 1, suppl.).

**Figure 8:**
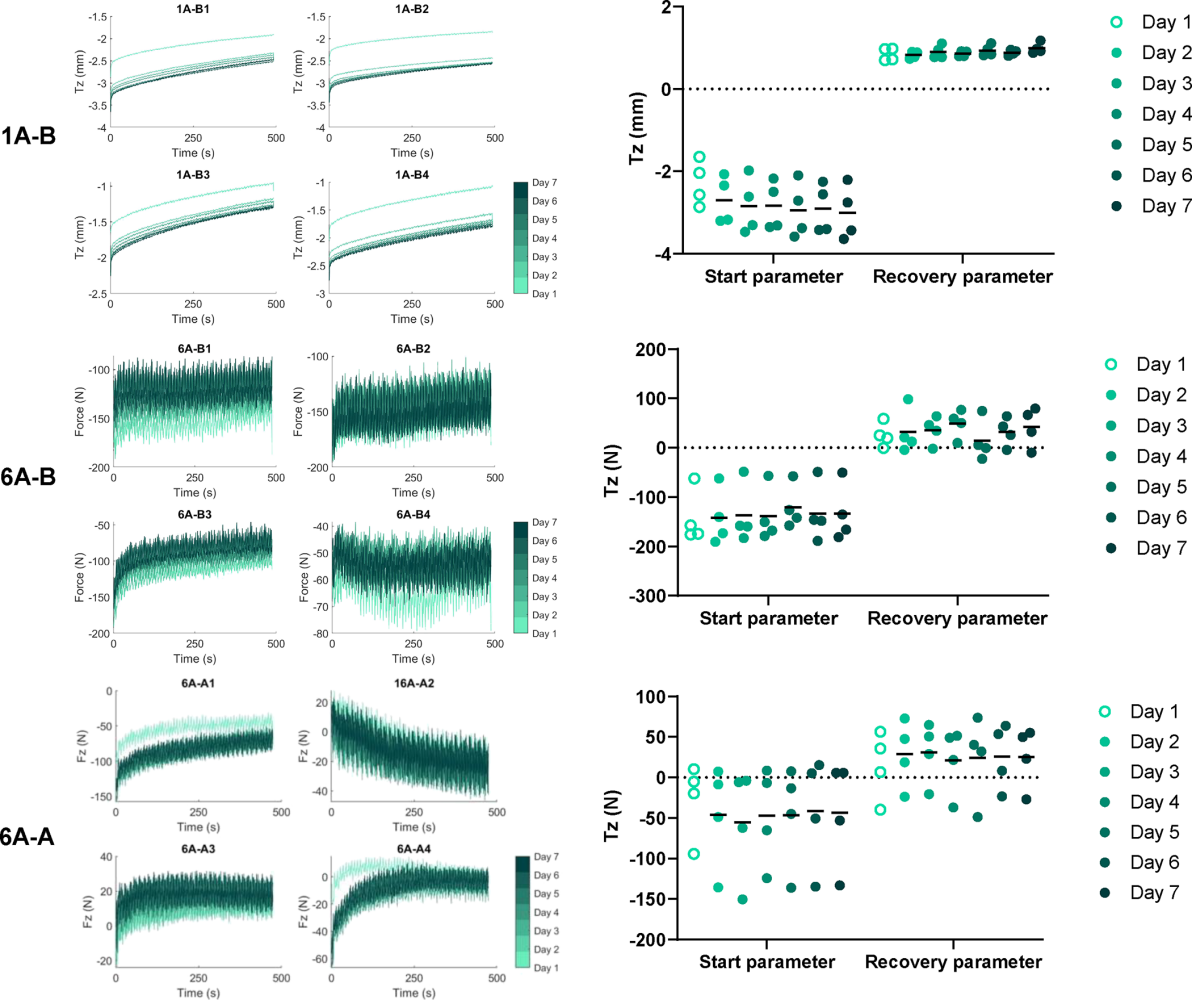
Recorded Fz translation (mm) in Tz for the single-axis UKBAP (1A-B1–4) and force (N) for both the six-axis UKBAP (6A-B1–4) and the six-axis active activity profile (6A-A1–4) throughout the sleeping period, over 7 days of testing (left panels). These data were used to determine the start and recovery parameters for 1A-B (top right), 6A-B (midright), and 6A-A (bottom right) groups, with lines denoting the mean value. It should be noted that the results for 1A-B should not be compared with 6A-B and 6A-A, as 1A-B outputs are the Tz position, whereas 6A-A and 6A-A outputs are the Tz force. Tz forces during the 6A-B and 6A-A profiles exhibit a higher level of noise compared to the Tz position; this reflects the six-axis profile, which replicates breathing and natural position changes during sleep, combined with load cell data inherently exhibiting more noise than the encoder signal used to measure the Tz position.

The ability of the IVD to withstand and recover from the daily loading regimes, which was evaluated using the disc height and axial force at the start of the sleep period (sleep start), and the change over the sleep period (sleep recovery) showed that the 1A-B group showed a nonsignificant reduction in IVD height identified via the sleep start, with a total reduction of 0.31 ± 0.14 mm from days 2 to 7, and although the sleep recovery increased by 0.16 ± 0.11 mm from days 2 to 7, this did not offset the slight reduction due to daily loading (Figure 8). This was not observed in the six-axis groups, and though these groups used kinematic control rather than the load control method of the 1A-B group, which increased interspecimen variability, it was observed that the change in axial compression (Tz) force used for the sleep parameters in the six-axis groups was relatively consistent from days 2 to 7 (Figure 8). The statistical analyses of the sleep parameters over the test period resulted in no significant differences in sleep start (*p* > 0.077) or sleep recovery (*p* > 0.244) in any of the loaded groups (1A-B, 6A-B, and 6A-A).

## Discussion

It is well-documented that the structures of the spine, including the IVD, are subjected to complex, si*x*-axis loads during ADLs.^32^ Activity monitoring^34^ and survey data^31^ have also shown that the type and intensity of those ADLs varies throughout the day. However, while previous IVD culture tests have demonstrated the large influence that loading can have on cell viability and gene expression,^8^ the loading regimes used have been highly simplified compared to *in vivo* loading.^25^ Therefore, this study reports the first use of a next-generation bioreactor for IVD research by successfully applying dynamic, six-axis, population-based activity profiles based on time use survey and *in vivo* ADL load data^28^ and biomechanical evaluation based on *in vivo* functional movement data from healthy participants.^29^ This multidisciplinary approach provides the first data of how different complex, dynamic, physiological loading regimes influence IVD health and answers previous calls to develop bioreactor systems capable of replicating the complex, six-axis loads that the human IVD is subjected to *in vivo*.^8,26,27^

By aiming to replicate the *in vivo* environment, the six-axis bioreactor facilitates detailed assessments of integration, biocompatibility, and mechanical performance of novel constructs, materials, and therapies. This is particularly advantageous in the development and preclinical evaluation of tissue-engineered and biomaterial strategies, as it bridges the gap between conventional *in vitro* studies and the complex dynamics of living tissues. The proposed system could offer critical insights into the efficacy and safety of innovative therapeutic approaches aimed at disc repair or regeneration during closely matched physiological conditions, thereby accelerating their development and speeding the process toward clinical translation.

The similar cell viability between D0 and D7 unloaded controls and the single-axis axial compression (1A-B) group align with previous research comparing unloaded and cyclic axial compression groups.^21^ However, lower cell viability with a static or dynamic axial compression compared to day 0 or unloaded controls^13,35,36^ and lower cell viability in unloaded groups compared to axially loaded groups^18^ have also been reported. These conflicting results may be due to factors relating to both specimen preparation and loading parameters across different studies. Almost all previous studies have entirely removed the vertebrae adjacent to the IVD to maintain nutritional pathways to the IVD, with some studies also removing the end plates.^15,16^ However, this may compromise the load transfer to and through the IVD, which could result in higher stresses and strains, leading to the observed differences in cell viability. The need to maintain some aspect of the adjacent vertebrae has been recognized for the application of multiaxis loads in IVD culture systems.^36^ A strength of the present study was the maintenance of IVD integrity through the inclusion of 10 mm of adjacent intact vertebra without interfering with the bone in contact with the end plates of the IVD. This provided the means to fix specimens in the biochamber and apply six-axis loading but will have also enabled the end plates, and the IVD as a whole, to withstand the axial compression that may have influenced cell viability in previous studies. However, in order to achieve this, it was necessary to adopt a specific preparation and culture system to minimize the risk of blood clots within the vertebra, so that endplate nutrition was not compromised.^30^ Previous bioreactor studies have demonstrated that increasing the axes used to load IVDs or the introduction of diurnal loading compared to static loading reduces cell viability.^15,21,23^ This suggests that more complex loading, which may better replicate *in vivo* loading conditions, creates a more challenging environment for IVD cells than zero or static load conditions or simplified loading regimes. This aligns with the findings of the present study in which the six-axis baseline activity profile (6A-B) resulted in significantly lower cell viability in both the NP and AF regions compared to unloaded controls (D0 and D7) and the single-axis activity profile (1A-B). This was further demonstrated with the more active si*x*-axis activity profile (6A-A) leading to an even lower cell viability in the AF region compared to the 6A-B group (Figure 4). The differential impact on NP and AF cell viability between the 6A-B and 6A-A profiles indicates that NP cells are more resilient to higher intensity of multiaxial loads compared to AF cells. This could be due to differences in the structural and functional roles of these regions, with the gelatinous structure of the NP being better able to absorb and distribute the stresses associated with ADLs. Finite element modeling has been used to calculate that AF cells experience transverse compressive strains two to five times higher than the ECM, whereas NP cells face smaller tensile, transverse, and compressive axial strains compared to the ECM.^37^ These differences suggest that AF cells endure more challenging mechanical conditions, which aligns with the higher-intensity six-axis profile having a greater impact on AF cell viability in the present study. However, while finite element modeling and other computational methods can provide a time and cost-effective way to explore how different loads and kinematics may influence disc structure and cell behavior, the validation of such models is critical if the results are to successfully aid our understanding of the IVD and provide a viable way to complete the *in silico* evaluation of new interventions. The results of the present study provide new data that can feed into the development and validation of such models, but it may be necessary to integrate further methods such as digital volume correlation,^38^ in order to understand how regional variations in tissue strain within the IVD may influence cellular behavior.

The only significant difference across different days of testing observed through the biomechanical evaluation occurred in LB in the 6A-A group, with no significant differences between day 2 and subsequent days identified through post hoc analyses. Overall, this demonstrated that there was good consistency in IVD biomechanics over the test period, suggesting that the test system and protocols were suitable for the maintenance of the IVD without introducing substantial structural damage or leading to IVD degradation.

Significant variations in the stiffness in axial compression throughout the day were observed in the single-axis group (1A-B). The simplified 1A-B loading regime compared to the si*x*-axis activity profiles, combined with the activity profiles being applied in load control, led to a much lower variability between specimens, which increased the ability to identify biomechanical changes across the day. The axial stiffness across all 1A-B tests of 686 ± 45 N/mm is comparable to previous bovine tail IVD compression tests to strains of 5–10%,^39^ based on ∼0.45 mm axial compressions during testing and bovine Cx1/2 IVD heights of 5.13 ± 0.83 mm measured previously via μCT imaging.^40^ However, the stiffness was often significantly lower in the morning compared to those in the afternoon and evening (Figure 5). This was likely due to the increased hydration and disc height following the sleep period (Figure 8), which aligns with diurnal changes *in vivo*, where the IVD height is significantly greater in the morning compared to the evening.^41^ Such clear time-of-day effects in axial stiffness were not observed in the six-axis test groups, though there were overall effects during FSB tests for both 6A-B and 6A-A groups, and during FBB tests in the 6A-A group. This is likely due to the nature of the tests completed. The biomechanical tests were based on the six-axis loads estimated from *in vivo* functional bending movements.^29^ Therefore, the effective axial stiffness was calculated from the change in axial force and position during each test, which would be influenced by the six-axis kinematics. The clearest time-of-day effect in the si*x*-axis groups was the axial rotation stiffness in the 6A-A group with a higher stiffness in the morning (Figure 7). Resistance to axial rotation is predominantly determined by a solid-phase behavior,^42^ with the expectation that it would be influenced more by the AF collagen structure than poroelastic effects due to fluid flow and changes in IVD hydration. However, a reduction in IVD height during the day may result in lower fiber tension in the AF leading to lower fiber strains during testing. As the AF tissue exhibits nonlinear stiffness,^43^ this would result in a lower measured stiffness in the evening. It is possible that this was observed only in the active 6A-A group because the diurnal changes in IVD height were less pronounced in the more sedentary 6A-B activity profile.

This study, while pioneering in its approach to replicating physiological load profiles on IVD specimens, has several limitations that merit consideration. Human population-based activity profiles were applied to bovine IVDs, which, despite being scaled based on IVD cross-sectional area,^28^ may be affected by differences between species. Although whole-organ IVD culture studies have been completed using human IVD specimens,^13,44^ the acquisition of healthy human IVD specimens soon after postmortem presents considerable barriers to making such tests routine. Bovine tail IVDs are similar to human IVDs with respect to compressive mechanical properties when normalized for geometric parameters,^45^ similar *in vivo* prone pressure (0.1–0.3 MPa),^46^ tissue composition,^45,47^ cell density,^47,48^ and the lack of notochordal cells in mature bovine IVDs,^47,48^ which and likely leads to the similar age-related declines in AF water content (bovine 67–73%, human 66–78%), although a difference is that human NP water content also decreases with age, which is not the case in bovine IVDs.^49^ Therefore, the overall similarities, along with the relative ease of access to healthy bovine tail IVD specimens, make them a highly relevant model that has been widely adopted for IVD bioreactor studies.^8^ However, potential remains that the six-axis activity profiles of the present study may lead to abnormal stresses and strains, and it would be valuable to explore adaptation periods or phased approaches to the activity profile application in the bovine tail IVD model.

Additionally, a critical challenge lies in the application of complex activity profiles to IVD specimens. Despite employing the Load Informed Kinematic Evaluation (LIKE) protocol to replicate *in vivo* loading,^28^ the use of kinematic control for the application of the six-axis activity profiles does not account for the inherent variability between specimens, which can lead to discrepancies in the actual loads applied. The use of the LIKE protocol imposes an instantaneous center of rotation (ICR) onto specimens based on previous six-axis load controlled tests of bovine tail IVD specimens,^28^ which may lead to differences between the natural ICR of a specimen, and that imposed by the six-axis bioreactor. Such differences may cause counteracting forces or moments, leading to the negative stiffness measurements reported in some of the biomechanical tests (Figures 7 and 8). This limitation was mitigated through the statistical methods used to evaluate the biomechanical outcome measures, which used repeated-measures two-way ANOVA, with time of day and day factors to identify changes in biomechanical parameters within each test group rather than direct comparisons of stiffness measurements. Another cause of such negative stiffness values could be misalignment of the specimen within the biochamber. An alignment jig was used to fix specimens onto the porous plates of the biochamber (Figure 2b), but a small amount of misalignment could influence movements that result in small load changes, such as axial force during axial rotation. These tests led to both positive and negative effective axial compression stiffness, but the mean across all specimens in both the 6A-B and 6A-A groups was close to the expected value of zero (Figures 7 and 8). Previous research has highlighted that small secondary rotations and translations occur, even during basic movements such as flexion and lateral bending, and that pure moment tests commonly used in biomechanical studies of the spine do not replicate physiological kinematics.^29^ Therefore, despite potential errors in loading magnitudes, replication of the complex six-axis kinematics of ADLs at physiological test rates does allow the application of more ecologically valid activity profiles for IVD whole-organ culture tests.

The D7 controls were incubated without being fixed to the porous plates and clamps that were required for the loaded groups (1A-B, 6A-B, and 6A-A). It is possible that the porous plates could have interfered with nutrition to the IVD. However, if that were the case, it would be expected that the single-axis group (1A-B) would have significantly different cell viability with respect to the unloaded controls (D0 and D7), which was not the case. This suggests that the custom porous plates used to mount the vertebrae of the loaded specimens along with the IVD-specific culture system did not lead to a compromise of IVD nutrition.

A further limitation concerns the scope of the investigation into IVD remodeling and longer-term responses to the activity profiles used in the present study. Analyses were limited to cell viability and biomechanical evaluation over a culture period of 7 days. While a culture period of 5–8 days is common in whole-organ IVD studies,^10,15−17,19,20,22^ longer culture periods that have also included intermediate time point analyses have shown that cell viability can increase following a decrease at day 7.^50,51^ Similarly, dynamic loading has led to anabolic remodeling in an *in vivo* dynamic loading rat model over 8 weeks,^52^ and hydration and glycosaminoglycan levels in the IVD and the IVD to vertebral height ratio, measured via MRI imaging in a human *in vivo* study, were significantly higher in long-distance runners compared to individuals that performed no regular sport or exercise.^53^ These studies highlight the adaptation and remodeling capability of the IVD, with the potential that the complex deformation patterns from dynamic loading can enhance the IVD resilience to repetitive and multidirectional stresses over time. The IVD stresses and strains when increasing physiological relevance, from unloaded controls and the single-axis activity profile, to the six-axis activity profiles, may have led to an increase in cell metabolism, which could explain the significantly lower cell viability over the 7-day culture period. This emphasizes the importance of integrating relevant loading regimes into bioreactor systems, and a focus of future research should be to complete longer-term investigations, along with the inclusion of gene expression and IVD composition analyses, and analyses at multiple time points, to more fully understand the extent and nature of IVD adaptations to the complex activity profiles used in the present study.

## Conclusions

The term “physiological loading” has been widely used in previous IVD bioreactor studies to define highly simplified loading protocols, which do not reflect the human *in vivo* environment. However, if IVD bioreactors are to be used to more fully understand human IVD mechanobiology and mechanisms of degeneration, evaluate regenerative interventions, and translate these findings into the clinical setting, it is critical that the loading conditions of the human IVD are replicated, which includes substantial periods of loading each day, loading in all six axes, and large ranges of loading magnitude and rate to reflect the diverse characteristics of different ADLs. Therefore, this study represents a significant advancement in the field, providing a novel means to investigate IVD biomechanics and cellular behavior under truly physiological conditions. The significant difference between the baseline (sedentary) and active six-axis activity profiles demonstrates the importance of considering a wide range of activities and lifestyles in IVD culture tests, and the ability to implement population-based activity profiles provides a valuable tool to explore the effects of lifestyle on IVD health, identify specific activities that promote anabolic remodeling to prevent IVD degeneration, and evaluate new devices and therapies for pathologies such as degenerative disc disease.

## Data Availability

The main data supporting the results in this study are available in the Article and Supporting Information. The raw and analyzed data sets generated during the study are available for research purposes from the corresponding author on reasonable request.

## Supporting Information Available

Statistics of repeated-measures two-way ANOVA for the effect of test day on disc stiffness (Supplementary data Table 1) (PDF)
Statistics of repeated-measures two-way ANOVA for the effect of time of day on disc stiffness (Supplementary Table 2) (PDF)
Six-axis kinematic control is achieved through an overall dSPACE controller, combined with individual motor controllers, a six-axis load cell and signal conditioning unit, and safety brakes. The dSPACE controller, and all individual motor controllers operate with a sample frequency of 10 kHz (Supplementary Figure S1) (PDF)
Step-by-step protocol used for the evaluation of cell viability in bovine tail IVDs (Supplementary Figure S2) (PDF)
Seven-day activity profile applied in the single-axis baseline group (1A-B) using load control in the axial compression axis (Fz) (Supplementary Figure S3) (PDF)
