# Supplementary material for: Six-Axis, Physiological Activity Profiles Create a More Challenging Cellular Environment in the Intervertebral Disc Compared to Single-Axis Loading
Source: ACS Biomater Sci Eng. 2025 Apr 23;11(5):3031–42. doi: 10.1021/acsbiomaterials.4c01773 (PMC12076284; doi:10.1021/acsbiomaterials.4c01773)
Supplement: Supplementary file 1 — ab4c01773_si_001.pdf [file ab4c01773_si_001.pdf]

Six-axis, physiological activity profiles create a more challenging cellular environment in the intervertebral disc compared to single-axis loading

*Daniela Lazaro-Pacheco[1] *, Isabelle Ebisch[1], Justin Cooper-White[2,3], Timothy P. Holsgrove[1]*

Note: Daniela Lazaro-Pacheco and Isabelle Ebisch are recognised as co-lead authors.

[1] Department of Engineering, Faculty of Environment, Science and Economy, University of Exeter, Harrison Building, Streatham Campus, North Park Road, Exeter, EX4 4QF, UK.

[2] School of Chemical Engineering, The University of Queensland, Australia.

[3] The UQ Centre in Stem Cell Ageing and Regenerative Engineering (StemCARE), Australian Institute for Bioengineering and Nanotechnology, The University of Queensland, Australia.

ORCID

DLP: https://orcid.org/0000-0002-0100-9416,

IE: https://orcid.org/0009-0000-7557-7849

JCW: https://orcid.org/0000-0002-1920-8229

TPH: https://orcid.org/0000-0003-2832-4958

.

Six-axis, physiological activity profiles create a more challenging cellular environment in the intervertebral disc compared to single-axis loading

Lazaro Pacheco, Daniela; Ebisch, Isabelle; Cooper-White, Justin; Holsgrove, Timothy

Supplementary Table 1. Statistics of repeated measures two-way ANOVA for the effect of test day on disc stiffness. Where test day was significant, p-values for post-hoc Dunnett's analysis are reported.

Group	Test	Day						
1A-B	FSB - AX	ANOVA	0.302					
	FBB - AX	ANOVA	0.319					
	LB - AX	ANOVA	0.360					
	AR - AX	ANOVA	0.220					
6A-B	FSB - ROT	ANOVA	0.656					
	FBB - ROT	ANOVA	0.624					
	LB - ROT	ANOVA	0.197					
	AR - ROT	ANOVA	0.418					
	FSB - AX	ANOVA	0.065					
	FBB - AX	ANOVA	0.204					
	LB - AX	ANOVA	0.136					
	AR - AX	ANOVA	0.491					
6A-A	FSB - ROT	ANOVA	0.441					
	FBB - ROT	ANOVA	0.404					
	LB - ROT	ANOVA	0.017					
		Post-hoc	Time of day	Day 2 vs day 3	Day 2 vs day 4	Day 2 vs day 5	Day 2 vs day 6	Day 2 vs day 7
			Afternoon	0.188	0.508	0.774	0.948	>0.999
			Evening	0.088	0.550	>0.999	0.677	0.638
			Morning	0.519	0.975	0.319	0.639	0.344
	AR - ROT	ANOVA	0.540					
	FSB - AX	ANOVA	0.059					
	FBB - AX	ANOVA	0.087					
	LB - AX	ANOVA	0.136					
	AR - AX	ANOVA	0.227					
