# Supplementary material for: Six-Axis, Physiological Activity Profiles Create a More Challenging Cellular Environment in the Intervertebral Disc Compared to Single-Axis Loading
Source: ACS Biomater Sci Eng. 2025 Apr 23;11(5):3031–42. doi: 10.1021/acsbiomaterials.4c01773 (PMC12076284; doi:10.1021/acsbiomaterials.4c01773)
Supplement: Supplementary file 2 — ab4c01773_si_002.pdf [file ab4c01773_si_002.pdf]

Six-axis, physiological activity profiles create a more challenging cellular environment in the intervertebral disc compared to single-axis loading

*Daniela Lazaro-Pacheco[1] *, Isabelle Ebisch[1], Justin Cooper-White[2,3], Timothy P. Holsgrove[1]*

Note: Daniela Lazaro-Pacheco and Isabelle Ebisch are recognised as co-lead authors.

[1] Department of Engineering, Faculty of Environment, Science and Economy, University of Exeter, Harrison Building, Streatham Campus, North Park Road, Exeter, EX4 4QF, UK.

[2] School of Chemical Engineering, The University of Queensland, Australia.

[3] The UQ Centre in Stem Cell Ageing and Regenerative Engineering (StemCARE), Australian Institute for Bioengineering and Nanotechnology, The University of Queensland, Australia.

ORCID

DLP: https://orcid.org/0000-0002-0100-9416,

IE: https://orcid.org/0009-0000-7557-7849

JCW: https://orcid.org/0000-0002-1920-8229

TPH: https://orcid.org/0000-0003-2832-4958

Six-axis, physiological activity profiles create a more challenging cellular environment in the intervertebral disc compared to single-axis loading

Lazaro Pacheco, Daniela; Ebisch, Isabelle; Cooper-White, Justin; Holsgrove, Timothy

Supplementary Table 2. Statistics of repeated measures two-way ANOVA for the effect of time of day on disc stiffness. Where time of day was significant, p-values for post-hoc Dunnett's analysis are reported.

Group	Test	Time of day				
1A-B	FSB - AX	ANOVA	0.006			
		Post-hoc	Day	Afternoon vs evening	Afternoon vs morning	Evening vs morning
			Day 2	0.559	0.015	0.058
			Day 3	0.943	0.001	0.002
			Day 4	0.028	0.013	0.073
			Day 5	0.644	0.029	0.094
			Day 6	0.782	0.031	0.067
			Day 7	0.907	0.057	0.041
	FBB - AX	ANOVA	0.007			
		Post-hoc	Day	Afternoon vs evening	Afternoon vs morning	Evening vs morning
			Day 2	0.571	0.018	0.044
			Day 3	0.605	0.001	0.001
			Day 4	0.000	0.014	0.039
			Day 5	0.784	0.042	0.103
			Day 6	0.599	0.037	0.057
			Day 7	0.982	0.054	0.043
	LB - AX	ANOVA	0.013			
		Post-hoc	Day	Afternoon vs evening	Afternoon vs morning	Evening vs morning
			Day 2	0.829	0.031	0.377
			Day 3	0.834	0.024	0.142
			Day 4	0.181	0.021	0.039
			Day 5	0.539	0.458	0.095
			Day 6	0.990	0.122	0.138
			Day 7	0.758	0.197	0.026
	AR - AX	ANOVA	0.002			
		Post-hoc	Day	Afternoon vs evening	Afternoon vs morning	Evening vs morning
			Day 2	0.741	0.025	0.231
			Day 3	0.947	0.029	0.065
			Day 4	0.153	0.007	0.015
			Day 5	0.209	0.146	0.032
			Day 6	0.533	0.031	0.023
			Day 7	0.617	0.072	0.010

Supplementary Table 2 continued

6A-B	FSB - ROT	ANOVA	0.664			
	FBB - ROT	ANOVA	0.635			
	LB - ROT	ANOVA	0.093			
	AR - ROT	ANOVA	0.196			
	FSB - AX	ANOVA	0.016			
		Post-hoc	Day	Afternoon vs evening	Afternoon vs morning	Evening vs morning
			Day 2	0.106	0.042	0.242
			Day 3	0.130	0.460	0.561
			Day 4	0.136	0.067	0.331
			Day 5	0.101	0.063	0.467
			Day 6	0.126	0.101	0.497
			Day 7	0.068	0.065	0.455
	FBB - AX	ANOVA	0.063			
	LB - AX	ANOVA	0.068			
	AR - AX	ANOVA	0.559			
6A-A	FSB - ROT	ANOVA	0.126			
	FBB - ROT	ANOVA	0.126			
	LB - ROT	ANOVA	0.439			
	AR - ROT	ANOVA	0.0317			
		Post-hoc	Day	Afternoon vs evening	Afternoon vs morning	Evening vs morning
			Day 2	0.032	0.182	0.060
			Day 3	0.070	0.125	0.037
			Day 4	0.041	0.149	0.043
			Day 5	0.033	0.175	0.052
			Day 6	0.052	0.166	0.039
			Day 7	0.036	0.155	0.042
	FSB - AX	ANOVA	0.028			
		Post-hoc	Day	Afternoon vs evening	Afternoon vs morning	Evening vs morning
			Day 2	0.145	0.338	0.100
			Day 3	0.121	0.654	0.097
			Day 4	0.137	0.409	0.099
			Day 5	0.138	0.536	0.123
			Day 6	0.092	0.394	0.066
			Day 7	0.100	0.456	0.071
	FBB - AX	ANOVA	0.028			
		Post-hoc	Day	Afternoon vs evening	Afternoon vs morning	Evening vs morning
			Day 2	0.143	0.297	0.089
			Day 3	0.132	0.569	0.098
			Day 4	0.178	0.383	0.099
			Day 5	0.137	0.447	0.098
			Day 6	0.104	0.354	0.061
			Day 7	0.141	0.401	0.078
	LB - AX	ANOVA	0.963			

	AR - AX	ANOVA	0.107
