# Supplementary material for: Six-Axis, Physiological Activity Profiles Create a More Challenging Cellular Environment in the Intervertebral Disc Compared to Single-Axis Loading
Source: ACS Biomater Sci Eng. 2025 Apr 23;11(5):3031–42. doi: 10.1021/acsbiomaterials.4c01773 (PMC12076284; doi:10.1021/acsbiomaterials.4c01773)
Supplement: Supplementary file 3 — ab4c01773_si_003.pdf [file ab4c01773_si_003.pdf]

Six-axis, physiological activity profiles create a more challenging cellular environment in the intervertebral disc compared to single-axis loading

Lazaro Pacheco, Daniela; Ebisch, Isabelle; Cooper-White, Justin; Holsgrove, Timothy

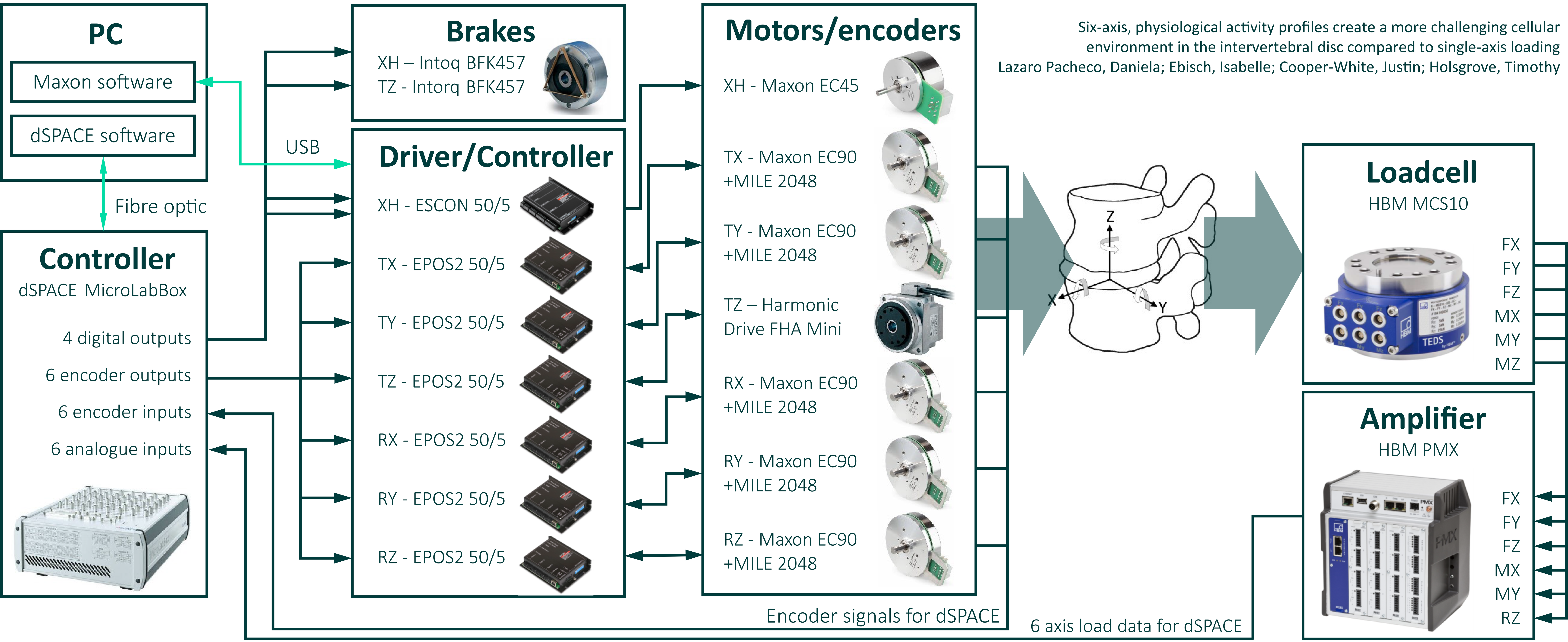

Supplementary Figure S1. Six-axis kinematic control is achieved through an overall dSPACE controller, combined with individual motor controllers, a six-axis load cell and signal conditioning unit, and safety brakes, as shown below. Both the dSPACE controller, and all individual motor controllers operate with a sample frequency of 10 kHz.
