# Supplementary material for: Six-Axis, Physiological Activity Profiles Create a More Challenging Cellular Environment in the Intervertebral Disc Compared to Single-Axis Loading
Source: ACS Biomater Sci Eng. 2025 Apr 23;11(5):3031–42. doi: 10.1021/acsbiomaterials.4c01773 (PMC12076284; doi:10.1021/acsbiomaterials.4c01773)
Supplement: Supplementary file 5 — ab4c01773_si_005.pdf [file ab4c01773_si_005.pdf]

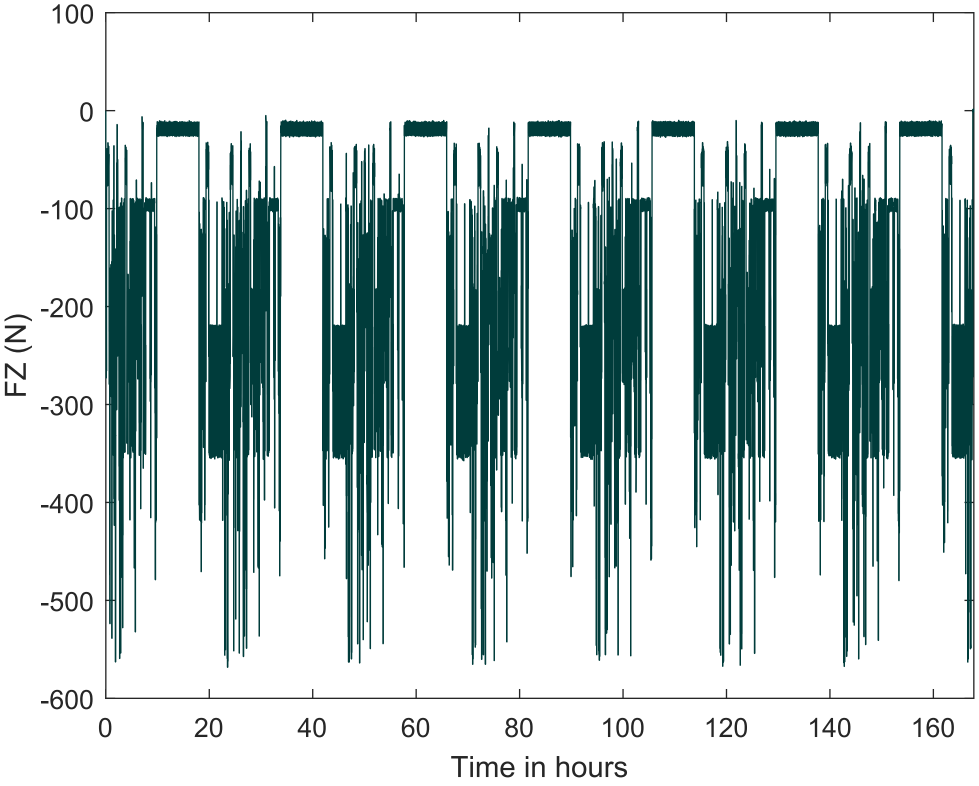

Supplementary Figure S3. The seven-day activity profile applied in the single-axis baseline group (1A-B) using load control in the axial compression axis (Fz).
